# Anthelmintic niclosamide suppresses transcription of *BCR-ABL* fusion oncogene via disabling Sp1 and induces apoptosis in imatinib-resistant CML cells harboring T315I mutant

**DOI:** 10.1038/s41419-017-0075-7

**Published:** 2018-01-22

**Authors:** Bei Jin, Chengyan Wang, Yingying Shen, Jingxuan Pan

**Affiliations:** 1 State Key Laboratory of Ophthalmology, Zhongshan Ophthalmic Center; Guangdong Provincial Key Laboratory of Brain Function and Disease, Sun Yat-sen University, Guangzhou, China; 2 Department of Pathophysiology, Zhongshan School of Medicine Sun Yat-sen University, Guangzhou, China; 3 Jinan University Institute of Tumor Pharmacology, College of Pharmacy, Jinan University, Guangzhou, China

## Abstract

Tyrosine kinase BCR-ABL fusion protein is the driver in patients with chronic myeloid leukemia (CML). The gate-keeper mutation T315I is the most challenging mutant due to its resistance to most tyrosine kinase inhibitors (TKIs). The third generation TKI ponatinib is the only effective TKI to treat CML patients harboring T315I-BCR-ABL mutation, but with high rate of major arterial thrombotic events. Alternative strategies to specifically target T315I-BCR-ABL are needed for the treatment of CML patients harboring such a mutation. Given that Sp1 is a fundamental transcriptional factor to positively regulate *WT-BCR-ABL* fusion oncogene, the purpose of this investigation was aimed at evaluating the anti-tumor activity and the underlying mechanism in terms of Sp1 regulational effect on the transcription of *T315I*-*BCR-ABL* fusion oncogene. Like in *WT-BCR-ABL*, we identified enrichment of Sp1 on the promoter of *T315I-BCR-ABL* fusion gene. Treatment of WT- and T315I-BCR-ABL-expressing CML cells by niclosamide diminished such an enrichment of Sp1, and decreased *WT- and T315I-BCR-ABL* transcription and its downstream signaling molecules such as STAT5 and Akt. Further, niclosamide significantly inhibited the proliferation and induced apoptosis through intrinsic pathway. The *in vivo* efficacy validation of *p*-niclosamide, a water soluble derivative of niclosamide, showed that *p*-niclosamide significantly inhibited the tumor burden of nude mice subcutaneously bearing T315I-BCR-ABL-expressing CML cells, and prolonged the survival of allografted leukemic mice harboring BaF3-T315I-BCR-ABL. We conclude that niclosamide is active against *T315I-BCR-ABL*-expressing cells, and may be a promising agent for CML patients regardless of T315I mutation status.

## Introduction

Chronic myeloid leukemia (CML) is a type of hematologic malignancy characterized by unrestrained expansion of myeloid leukemia cells in bone marrow and generally progresses from chronic phase (CP), to accelerated phase and then blast phase^1,2^. The cytogenetic hallmark of the disease is the presence of a reciprocal chromosomal translocation t(9; 22) (q34; q11) resulting in a derivative 9q^+^ and a small 22q^-^, known as the Philadelphia (Ph) chromosome^3^. Ph chromosome harbors the *BCR-ABL* fusion oncogene encoding the deregulated tyrosine kinase BCR-ABL chimeric protein, which is necessary and sufficient for the transformed phenotype of CML cells^4–7^. BCR-ABL can activate downstream signaling pathways such as STAT5, PI3K/Akt, and Erk1/2 to lead to increased cell transformation, survival, and proliferation^8–12^. TKI imatinib mesylate markedly improves survival of patients with CP-CML. However, acquired resistance to imatinib can develop, giving rise to disease relapse and progression^13^. Resistance to imatinib is attributed to multiple mechanisms. For instance, acquisition of point mutations in *BCR-ABL* gene (e.g., T315I, F317L, F359C/V, G250E, Q252H, and E255K/V) accounts for ~50% of imatinib-resistance cases^7,14,15^. Other factors may involve existence of quiescent CML stem cells^16–19^, overexpression of SRC family of kinases^20^ and LYN kinase^21^, and binding of imatinib by α1-acid glycoprotein^22^.

Acquisition of BCR-ABL mutations directly or indirectly altering the protein conformation, resulting in poor adherence are the most frequent cause of treatment failure and imatinib-resistance^7,23^. Most of the identified imatinib-resistant BCR-ABL mutants but T315I are sensitive to the second generation TKIs nilotinib and dasatinib. The gate-keeper mutation T315I is the most challenging mutant due to its vicious resistance to multiple TKIs^24^. Although approved by the US Food and Drug Administration (FDA) for the treatment of CML patients harboring T315I-BCR-ABL mutation^25^, the third generation of TKI ponatinib encounters high rate of major arterial thrombotic and life-threatening side-effect events^26^. Therefore, alternative strategies or novel drugs targeting the T315I-BCR-ABL mutant are urgently needed for the treatment of CML patients harboring such a mutation.

Blockade of oncogene transcription is an attractive approach to abrogate oncogene addiction and overcome drug-resistance. In the context of *BCR-ABL* oncogene, its transcription is positively regulated by transcription factor Sp1. Silencing Sp1 can diminish *BCR-ABL* expression and abolish its downstream signaling^27^. However, whether Sp1 regulates *T315I-BCR-ABL* mutant oncogene remains elusive.

Niclosamide, an FDA-approved anthelmintic, has been used to treat tapeworm infection for about 50 years^28^. Several studies revealed that niclosamide have inhibitory effects on multiple overexpressed or constitutively active intracellular signaling pathways in various cancer cells, rendering niclosamide as a potential anticancer agent. These pathways include Wnt/β-catenin^29,30^, STAT3^31,32^, and Notch^33^. Previous report from us showed that niclosamide inactivates the NF-κB pathway and kills progenitor/stem cells from AML patients^34^. Recently, our group has demonstrated that niclosamide can eradicate leukemia stem cells (LSCs) in CML through disrupting interaction between p65 and FOXM1/β-catenin^18^, suggesting its activity against imatinib-resistance caused by LSCs. Whereas, whether niclosamide is active against mutational resistance caused by *T315I-BCR-ABL* remains to be explored.

Given that Sp1 is a fundamental transcriptional factor to positively regulate *WT-BCR-ABL* fusion oncogene, the purpose of this investigation was aimed at evaluating the anti-tumor activity and the underlying mechanism in terms of Sp1 regulational effect on the transcription of *T315I*-*BCR-ABL* fusion oncogene. Like in *WT-BCR-ABL*, we identified enrichment of Sp1 on the promoter of *T315I-BCR-ABL* fusion gene. Treatment of WT- and T315I-BCR-ABL-expressing CML cells by niclosamide diminished such a enrichment of Sp1, and decreased WT- and T315I-BCR-ABL transcription and its downstream signaling molecules such as STAT5 and Akt. We also validated the *in vivo* efficacy of niclosamide in two different mouse models.

## Results

### Niclosamide inhibits expression of WT- and T315I-BCR-ABL at transcriptional level

**Fig. 1:**
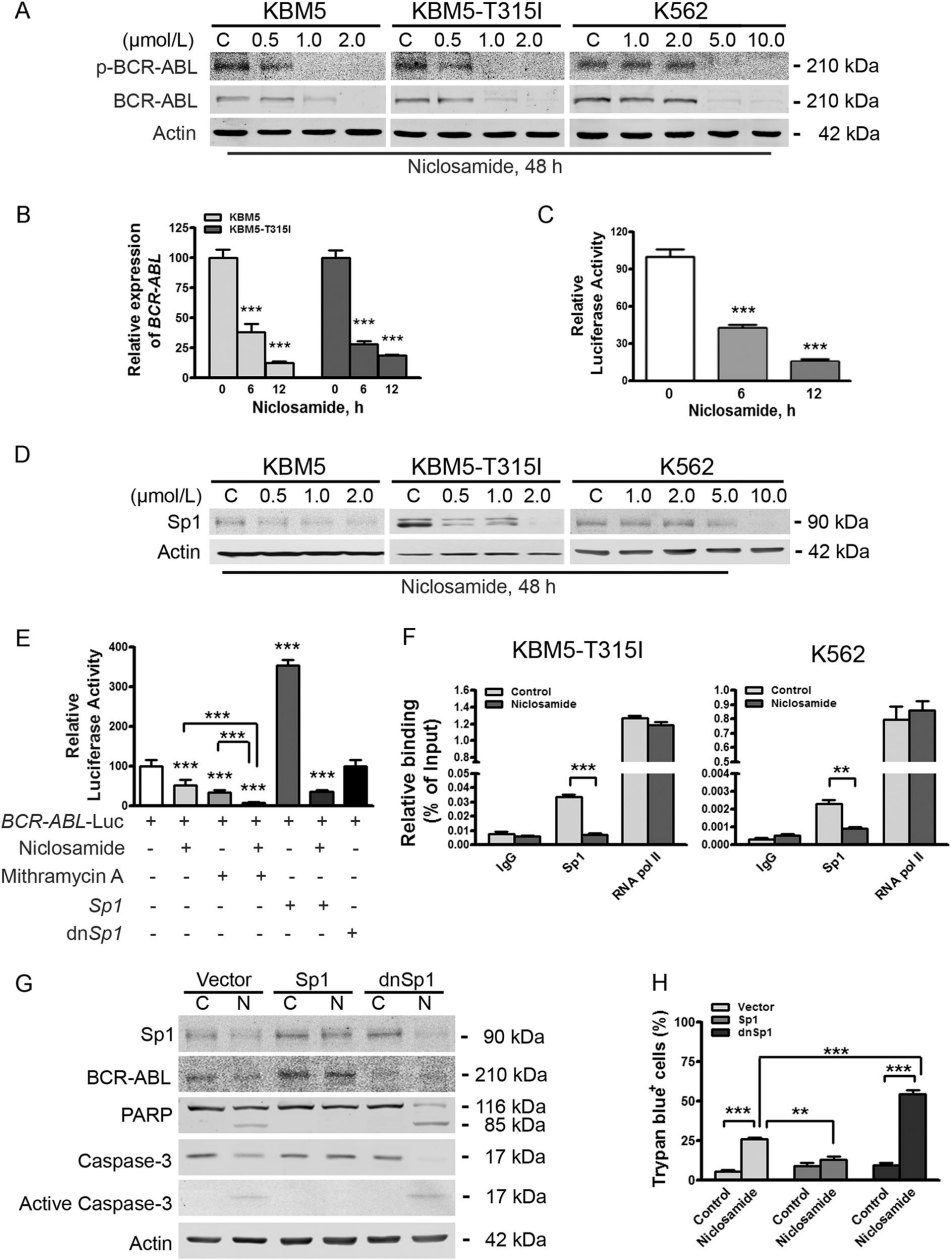
Niclosamide suppresses transcription of *BCR-ABL* gene by lowering transcriptional factor Sp1 in CML cells harboring either wild-type- or T315I-BCR-ABL **a** KBM5 cells harboring wild-type or T315I-BCR-ABL and K562 cell were exposed to different concentrations of niclosamide, and then analyzed by Western blotting analysis. **b** KBM5 and KBM5-T315I cells were treated with or without niclosamide (2.0 μmol/L) for 6 or 12 h, and then underwent qRT-PCR analysis for *BCR-ABL* gene. ***P < 0.0001, compared with control, one-way ANOVA, *post hoc* intergroup comparisons. **c** Twenty-four hours after transfected with plasmids encoding *BCR-ABL* gene promoter-Luc and *Rellina*-Luc, 293T cells were treated with or without 2.0 μmol/L niclosamide for the indicating durations, followed by dual-luciferase reporter assay. ***P < 0.0001, compared with control, one-way ANOVA, *post hoc* intergroup comparisons. **d** Sp1 levels were downregulated in CML cells. KBM5, KBM5-T315I, and K562 cells were treated with concentrations of niclosamide for 48 h and subjected to Western blotting analysis. **e** Sp1 promoted the transcription of *BCR-ABL* gene. 293T cells were transfected with *BCR-ABL* gene promoter-Luc, *Rellina*-Luc, and *Sp1* or dn*Sp1*, then exposed to 2.0 μmol/L niclosamide for 6 h and/or 0.4 μmol/L mithramycin A for 24 h, and analyzed by dual-luciferase activity assay. ***P < 0.0001, compared with control, one-way ANOVA, *post hoc* intergroup comparisons. **f** Niclosamide inhibits the enrichment of Sp1 on the promoter *of BCR-ABL* and T315I-*BCR-ABL*. K562 and KBM5-T315I cells were incubated with or without niclosamide (5.0 μmol/L for K562, 2.0 μmol/L for KBM5-T315I) for 24 h, cell lysates were subjected to ChIP assay for qPCR analysis of ChIP products. **g**, **h** KBM5-T315I cells were transfected with plasmids of vector, Sp1 or dnSp1, treated with control (C) or 2.0 μmol/L niclosamide (N) for 24 h, and then subjected to Western blotting analysis (**g**) and trypan blue exclusion assay (**h**)

We first determined the effect of niclosamide on BCR-ABL in CML cells. KBM5, KBM5-T315I, and K562 cells were incubated with niclosamide at increasing concentrations for 48 h. Western blotting analysis showed that the total protein levels of either WT- or T315I-BCR-ABL were decreased in a concentration-dependent manner (Fig. 1a). Correspondingly, the levels of phospho-BCR-ABL and phospho-T315I-BCR-ABL were declined (Fig. 1a). Similarly, niclosamide elicited downregulation of WT- or T315I-BCR-ABL protein in a time-dependent manner (Supplementary Fig. S1A).

We next asked whether the mRNA levels of WT- and *T315I*-*BCR-ABL* were changed by niclosamide. After incubated with niclosamide, KBM5 and KBM5-T315I cells were subjected to real-time RT-PCR analysis. The levels of *BCR-ABL* mRNA were significantly reduced (Fig. 1b). Alternatively, 293T cells were transfected with plasmids encoding *BCR-ABL* gene promoter-Luc and *Rellina*-Luc, and exposed to niclosamide for 6 or 12 h; dual-luciferase reporter assay indicated that niclosamide inhibited the transcription activity of *BCR-ABL gene* (Fig. 1c). We, therefore, conclude that niclosamide treatment leads to downregualtion of BCR-ABL expression at transcriptional level.

### Niclosamide suppresses transcription of *BCR-ABL* gene in a Sp1-dependent manner even in T315I-BCR-ABL-positive CML cells

The expression of *BCR-ABL* oncogene is positively regulated by transcription factor Sp1^27^. To investigate whether the decrease of BCR-ABL levels by niclosamide was mediated by Sp1, we assessed the effect of niclosamide on the expression levels of Sp1 by Western blotting analysis. The results showed that the levels of Sp1 in KBM5, KBM5-T315I, and K562 cells were obviously declined by niclosamide in a concentration- and time-dependent manner (Fig. 1d and Supplementary Fig. S1b). These results indicate that Sp1 may play a vital role in the downregulation of BCR-ABL by niclosamide.

We next examined the impact of Sp1 on *BCR-ABL* gene expression. 293T cells were co-transfected with constructs encoding *BCR-ABL* gene promoter-Luc and *Sp1* or dominant negative *Sp1* (dn*Sp1*). Luciferase activity analysis revealed that Sp1 significantly promoted the transcription of *BCR-ABL* fusion gene (Fig. 1e). In contrast, dn*Sp1* exerted minimal stimulating effect on the transcription of *BCR-ABL* fusion gene (Fig. 1e). When the 293T cells co-transfected with constructs encoding *BCR-ABL* gene promoter-Luc and and constructs encoding *Sp1* were exposed to niclosamide, the luciferase activity of *Sp1* promoter-Luc was remarkably abrogated. These data suggest that Sp1 mediates *BCR-ABL* transcription, which can be effectively blocked by niclosamide treatment.

Considering the presence of cellular endogenous Sp1, 293T cells transfected with constructs encoding *BCR-ABL* gene promoter-Luc were treated with mithramycin A (MMA), a selective inhibitor of Sp1, or in combination with niclosamide for 24 h. Luciferase activity analysis indicated that treatment of MMA significantly decreased the luciferase activity of *BCR-ABL* gene promoter, which is in line with the finding that the exogenous Sp1 increases *BCR-ABL* transcription. Combinational treatment between MMA and niclosamide elicited an enhanced decrease in the luciferase activity of *BCR-ABL* gene promoter (Fig. 1e), suggesting that niclosamide may inhibit the transcription of *BCR-ABL* gene at least partially in a Sp1-dependent manner.

Further, results of ChIP assay showed an enrichment of Sp1 on the promoter of either WT- or T315I-*BCR-ABL* gene, which was sensitive to niclosamide treatment (Fig. 1f).

### Sp1 is critical for BCR-ABL expression and niclosamide-mediated apoptosis in KBM5-T315I cells

To further confirm the positively regulatory role of Sp1 on BCR-ABL expression, KBM5-T315I cells were transfected with empty vector or plasmids encoding Sp1 or dnSp1. Levels of endogenous BCR-ABL were elevated in the Sp1-overexpressed CML cells, but declined in the dnSp1-overexpressed CML cells (Fig. 1g). To examine the relative contribution of Sp1 in niclosamide-induced apoptosis, KBM5-T315I cells with empty vector, Sp1 or dnSp1 were exposed to control culture medium or niclosamide for 24 h. The cells with empty vector underwent moderate apoptosis, whereas *Sp1-* encoding constructs-transfected cells exhibited no increase in apoptosis, as indicated by specific cleavage of PARP, activation of caspase-3, and trypan blue exclusion assay (Fig. 1g, h). Conversely, the cells with dnSp1 extensively potentiated the capability of niclosamide to induce apoptosis. However, silencing of Sp1 alone was not sufficient to trigger apoptosis. Therefore, Sp1 might play a vital role in niclosamide-mediated apoptosis.

### Niclosamide blocks BCR-ABL downstream signaling but Erk1/2

**Fig. 2:**
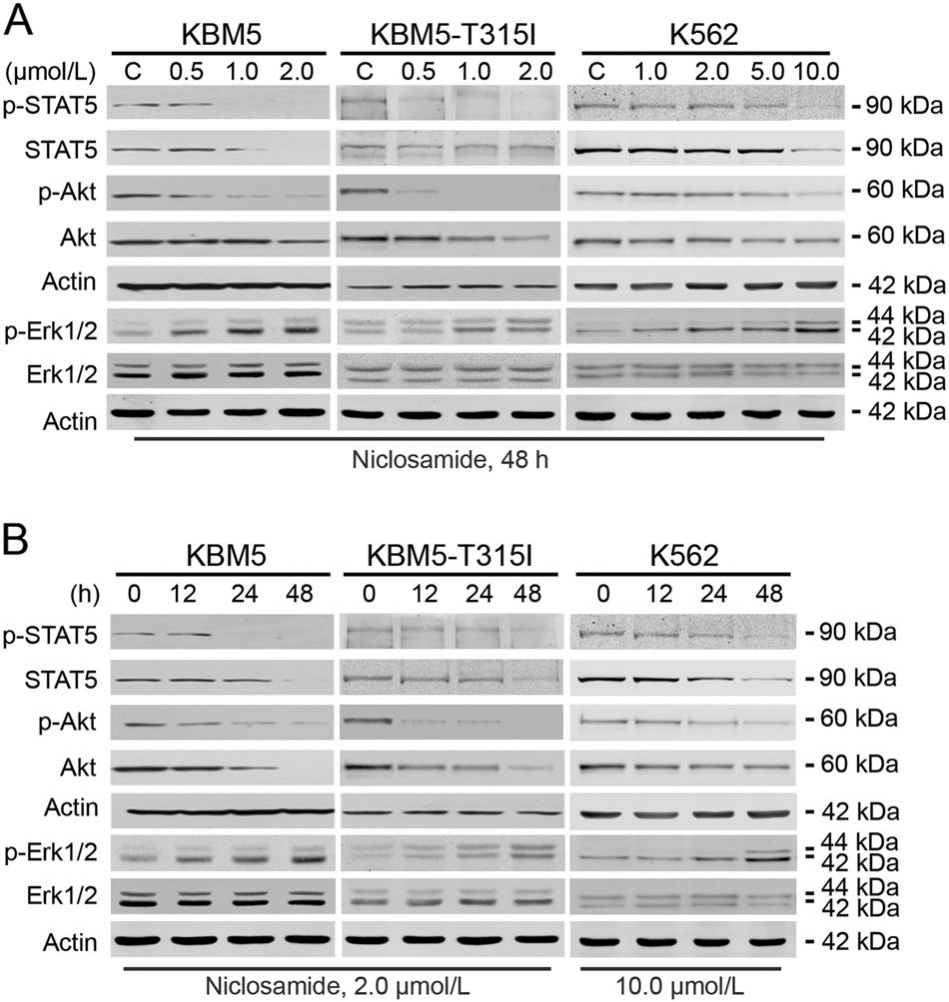
Niclosamide inhibits the downstream molecules of BCR-ABL CML cells were treated with increasing concentrations of niclosamide for 48 h (**a**) or a fixed concentration for different durations (**b**) and subjected to Western blotting analysis. Actin served as a loading control for blots above, which were performed sequentially

Because the levels of phospho-BCR-ABL and total BCR-ABL were decreased by niclosamide, we next examined the downstream signaling. After treatment with niclosamide, the phosphorylated and total protein levels of STAT5 and Akt were remarkably decreased in a concentration- and time-dependent manner in KBM5 and KBM5-T315I cells (Fig. 2a, b). In contrast, the levels of phospho-Erk1/2 (T202/Y204) were elevated in a dose- and time-dependent manner although minimal change in the total Erk1/2 protein was observed (Fig. 2a, b), which might be caused by mTORC1-MAPK feedback loop^35^, and raise a potential risk of resistance to niclosamide.

### Niclosamide inhibits the growth of T315I-BCR-ABL-harboring cells

**Fig. 3:**
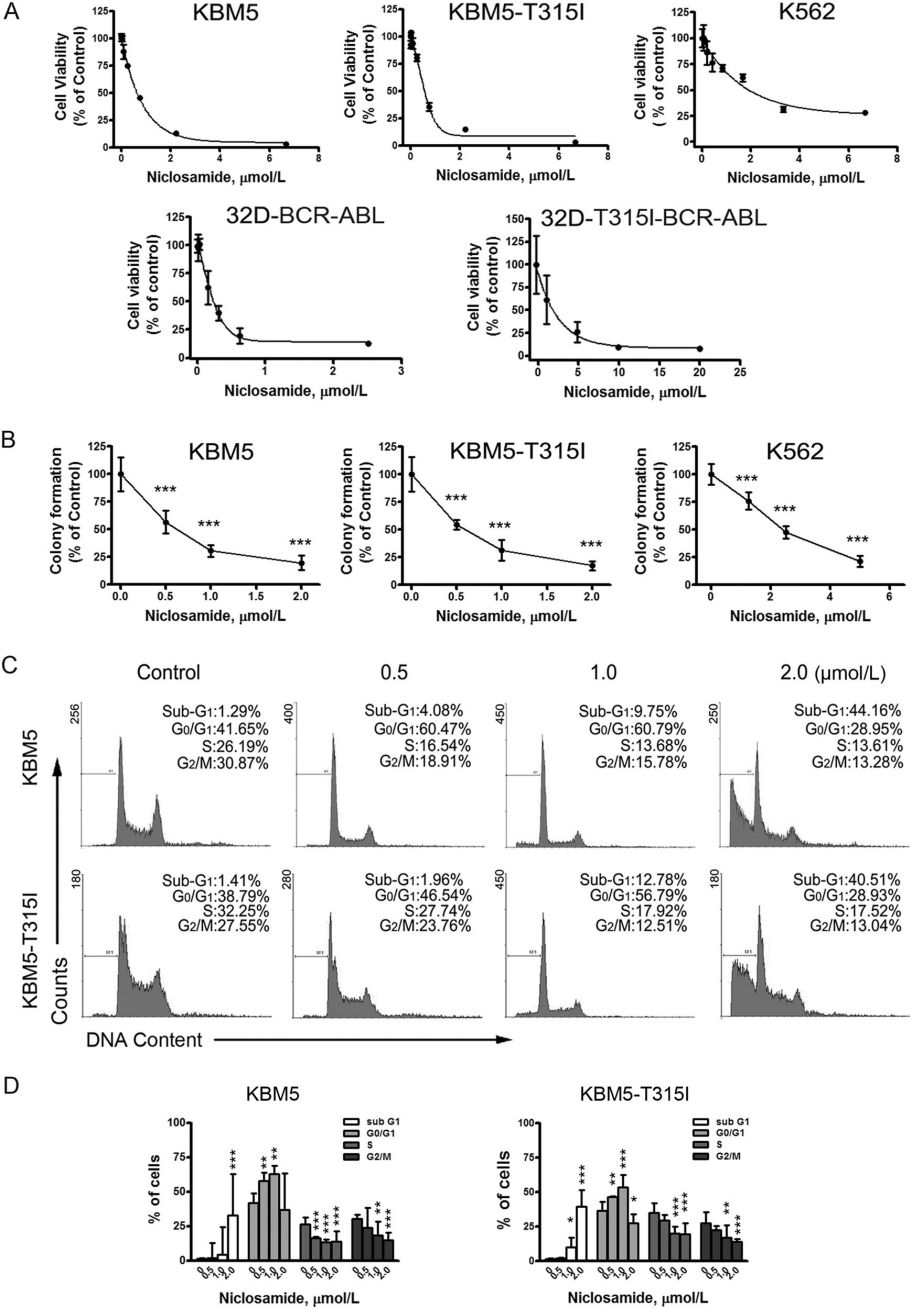
Niclosamide inhibits cellular proliferation in CML cells **a** Cell viability in KBM5, KBM5-T315I, and K562 cells was assessed by MTS assay after 72 h treatment of different concentrations of niclosamide. Data are mean ± 95% confidence intervals from three independent experiments. **b** Clonogenicity of KBM5, KBM5-T315I, and K562 cells in soft agar was inhibited by niclosamide in a dose-dependent manner. ***P < 0.0001, compared with control, one-way ANOVA, *post hoc* intergroup comparisons. **c**,** d**, KBM5 cells harboring wild-type- or T315I-BCR-ABL were treated with niclosamide for 24 h and stained with propidium iodide (PI) for flow cytometer analysis. Histogram data were from a set of representative experiments (**c**). Bar charts of the cell cycle phases from three independent experiments were shown (**d**). *P < 0.05, **P < 0.001, ***P < 0.0001, compared with control, one-way ANOVA, *post hoc* intergroup comparisons

We next determined the effect of niclosamide on growth of CML cells. A set of CML cell lines harboring the imatinib-resistant mutation or not, were incubated with niclosamide for MTS assay. Cell viability was extremely inhibited with IC_50_ values of 0.65 μmol/L, 0.58 μmol/L, and 2.31 μmol/L in KBM5, KBM5-T315I, and K562 cells, respectively (Fig. 3a, top). For 32D-BCR-ABL and 32D-T315I-BCR-ABL cells, IC_50_ values were 0.33 μmol/L and 3.06 μmol/L, respectively (Fig. 3a, bottom). Separately, colony-formation assay results showed that niclosamide potently inhibited the clonogenicity in a dose-dependent manner (Fig. 3b). The IC_50_ values were 0.92 µmol/L, 0.89 µmol/L, and 6.13 µmol/L, respectively.

To investigate if niclosamide inhibited cell growth through cell cycle arrest, we labeled KBM5 and KBM5-T315I cells with propidium iodide (PI) for cytometery analysis. Cell cycle distribution analysis showed that low concentrations of niclosamide induced G_0_/G_1_-phase arrest, while high concentrations niclosamide increased substantial sub G_1_ proportion, which is indicative of cell death (Fig. 3c, d)^36^.

### Niclosamide induces apoptosis in T315I-BCR-ABL-harboring cells

**Fig. 4:**
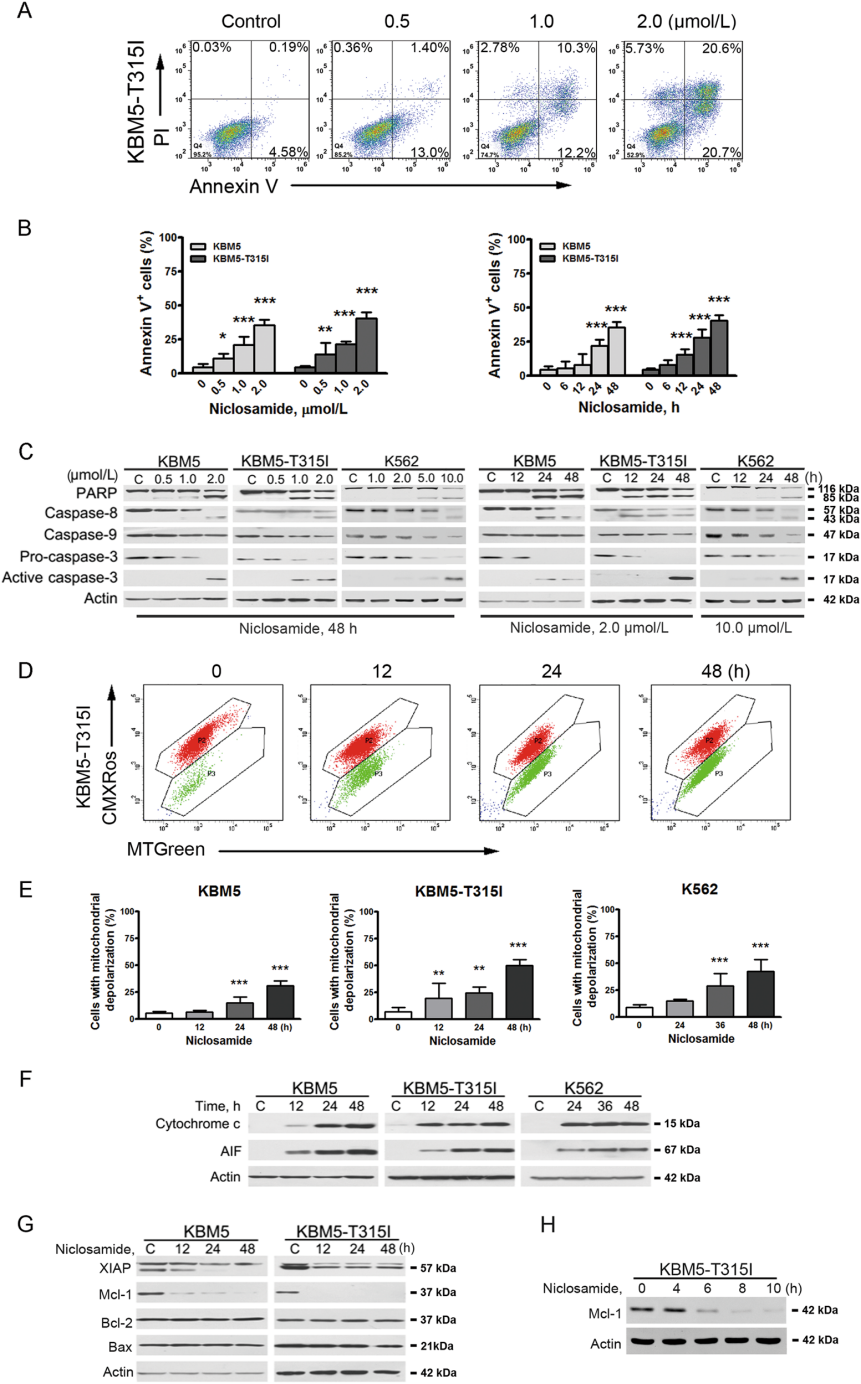
Niclosamide induces apoptosis in CML cells through intrinsic pathway **a**, **b** KBM5 and KBM5-T315I cells were treated with niclosamide at the indicating concentrations for 48 h or with 2.0 μmol/L niclosamide for various durations, then labeled with annexin V-FITC and PI for flow cytometer analysis. A set of representative dot plots of KBM5-T315I flow cytometer analysis were shown (**a**). Bar charts with mean ± 95% confidence intervals from three independent experiments were shown (**b**). The *y*-axis was the sum of top right and bottom right quadrants. **P < 0.01; ***P < 0.0001, one-way ANOVA, compared with control, *post hoc* intergroup comparisons. **c** Dose-dependent and time-course Western blotting analysis of PARP cleavage, the activation of caspase-3, -8, and -9 in the whole-cell lysates of human CML cells were shown. **d**, **e** After treated with niclosamide for different durations, KBM5, KBM5-T315I, and K562 cells were stained with CMXRos and MTGreen, then subjected to flow cytometer analysis for mitochondrial potential. A set of representative plot of KBM5-T315I cells (**d**) and bar charts with mean ± 95% confidence intervals from three independent experiments (**e**) were shown. Vertical axis represents the cell population of region P3. **P < 0.01; ***P < 0.0001, compared with control, one-way ANOVA, *post hoc* intergroup comparisons. **f** Niclosamide induced the release of apoptosis-inducing factor (AIF) and cytochrome *c* from the mitochondria. KBM5, KBM5-T315I, and K562 cells were treated with 2.0 μmol/L, 2.0 μmol/L, and 10.0 μmol/L niclosamide for the indicating time, respectively. **g**, **h** Niclosamide decreased the level of Mcl-1 and XIAP in a time-dependent manner. KBM5 and KBM5-T315I cells with 2.0 µmol/L niclosamide for different durations were subjected to Western blotting analysis

We next evaluated the capability of niclosamide to induce apoptosis. CML cells harboring WT- and T315I-BCR-ABL were treated with niclosamide followed by dual staining of annexin V-FITC/PI for flow cytometer analysis. The results showed that increased cell death in CML cells were induced by niclosamide in a concentration- and time-dependent manner (Fig. 4a, b). Further, niclosamide concentration- and time-dependently induced specific cleavage of PARP and caspase-8, -9, and -3 (Fig. 4c). These results further indicate the occurrence of apoptosis in CML cells including those harboring imatinib-resistant T315I-BCR-ABL.

### Niclosamide elicits mitochondrial damage in T315I-BCR-ABL-harboring cells

We assessed the mitochondrial potential by flow cytometer after staining with CMXRos and MTGreen. The results revealed that niclosamide induced a marked mitochondrial depolarization in all tested three lines of CML cells (Fig. 4d, e). We next detected the release of cytochrome *c* and AIF. CML cells incubated with niclosamide for different durations, levels of cytochrome *c* and AIF in the cytosolic fraction were increased after niclosamide treatment (Fig. 4f), which was prior to the activation of caspase-9 and caspase-3. These results indicate that niclosamide might give rise to mitochondria damage and trigger the intrinsic pathway of apoptosis in CML cells.

### Niclosamide leads to downregulation of Mcl-1 and XIAP

To clarify the mechanism of niclosamide-induced apoptosis, we assessed the expression of apoptosis-related proteins. Western blotting analysis in KBM5 and KBM5-T315I cells revealed a decrease in XIAP and Mcl-1 in a concentration- and time-dependent fashion, with no alteration in Bcl-2 and Bax (Fig. 4g and Supplementary Fig. S2). Time-course observation of Mcl-1 by immunoblotting revealed an extensive decline as early as 6 h after niclosamide treatment (Fig. 4h), which preceded the appearance of considerable accumulation of Annexin V^+^ cells as indicated in Fig. 4b. These results suggest that decrease of Mcl-1 may be a trigger of niclosamide-induced apoptosis.

### Niclosamide is synergistic with TKIs and MEK inhibitor

**Fig. 5:**
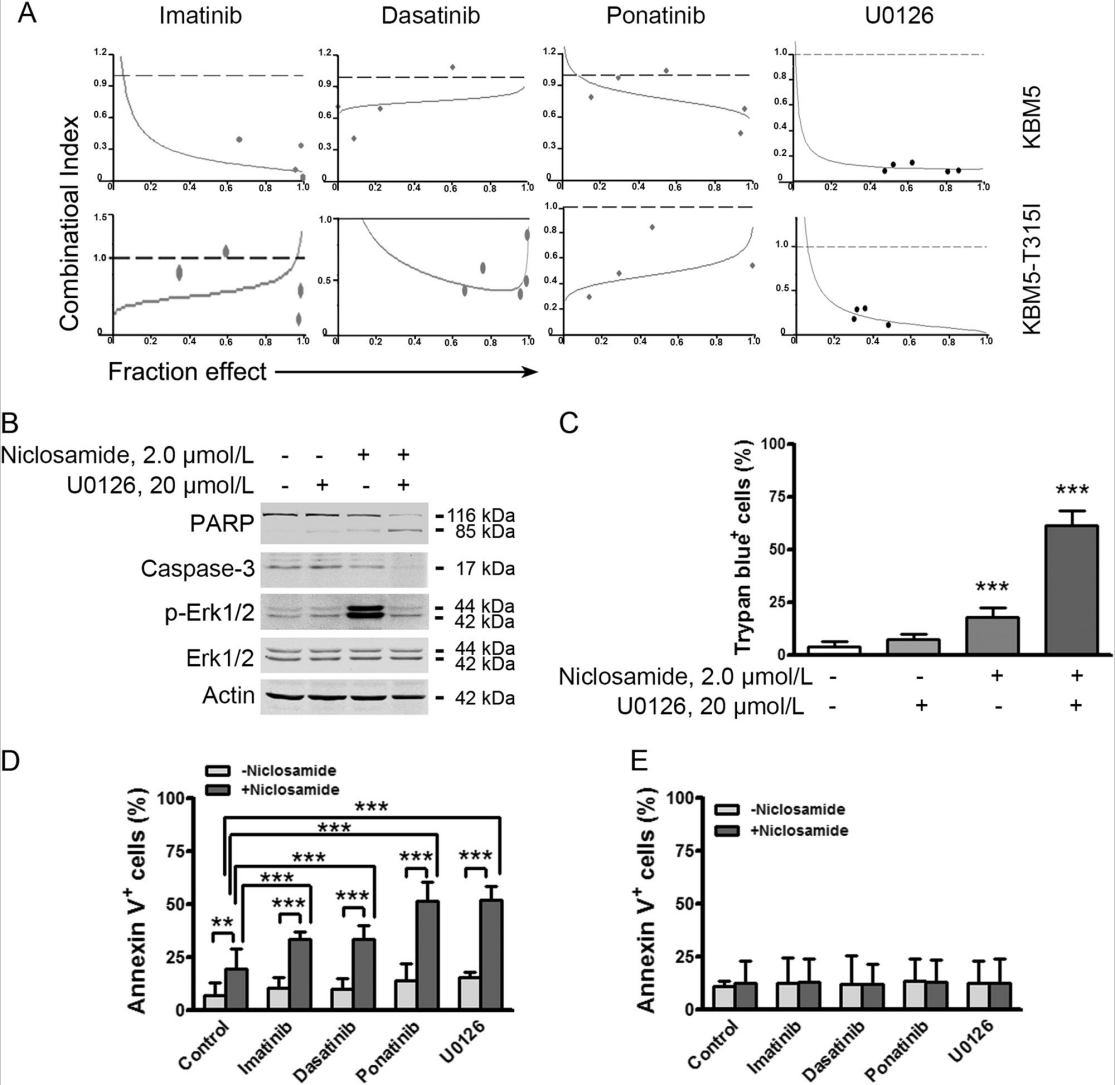
Niclosamide is synergistic with tyrosine kinase inhibitors and MEK inhibitor **a** Niclosamide was synergistic with tyrosine kinase inhibitors (imatinib, dasatinib, and ponatinib) and MEK inhibitor (U0126) in CML cells. Synergistic effect of the combination of niclosamide and tyrosine kinase inhibitors or MEK inhibitor in CML cells was assessed by MTS assay after incubating cells with a serially diluted mixture at a fixed ratio of the two drugs. CI is plotted against the fraction effect. The reference line indicates CI = 1. CI value below 1 indicates synergism between the two drugs. **b**, **c** Niclosamide combined with U0126 induced an enhanced apoptosis in CML cells. KBM5-T315I cells were treated with niclosamide, U0126 or the combination as indicated for 24 h, the cells were then harvested for Western blotting analysis (**b**) and trypan blue exclusion assay (**c**). Actin served as a loading control for blots above which were performed sequentially. ***P < 0.0001, compared with control, one-way ANOVA, *post hoc* intergroup comparisons. **d, e** KBM5-T315I cells (**d**) and normal WBC (**e**) were treated with control medium, niclosamide (2.0 µmol/L) combined with imatinib (0.50 µmol/L), dasatinib (0.10 µmol/L), ponatinib (0.02 µmol/L), or U0126 (20.0 µmol/L) for 24 h, followed by annexin V-FITC/PI dual staining and flow cytometer analysis

We next examined the potential synergism between niclosamide and the clinically approved TKIs (e.g., imatinib, dasatinib, and ponatinib) in growth suppression of CML cells. KBM5 and KBM5-T315I cells were incubated in a serially diluted mixture of niclosamide and imatinib, dasatinib or ponatinib at a fixed ratio for 72 h, and subjected to MTS assay. Synergism was evaluated by the median-effect method of Chou and Talalay^37^. Niclosamide showed synergism with imatinib, dasatinib, and ponatinib as indicated by combination index (CI) < 1 (Fig. 5a).

Because the elevated levels of phospho-Erk1/2 (T202/Y204) after treatment of niclosamide may raise a potential risk of resistance to niclosamide (Fig. 2), we examined the combined therapeutic approach with niclosamide and MAPK inhibitors. KBM5-T315I cells were exposed to niclosamide ± U0126. Western blotting analysis revealed an apparent blocking effect of Erk1/2 activity by U0126, and an enhanced cleavage of PARP in the CML cells treated with combination (Fig. 5b). Similarly, trypan blue exclusion assay revealed a robust cell death in KBM5-T315I cells treated with combination (Fig. 5c), comparing with either niclosamide or U0126 alone. These data verified the combined therapeutic approach with niclosamide and MEK inhibitor.

The combination effects of niclosamide with TKIs on cell death in KBM5-T315I cells and healthy peripheral white blood cells (WBC) were further verified. The results showed that niclosamide induced substantial cell death when combined with imatinib, dasatinib, ponatinib or U0126 in KBM5-T315I cells (Fig. 5d), while sparing the healthy WBC (Fig. 5e).

### *p*-Niclosamide inhibits the growth of xenografted T315I-BCR-ABL cells in nude mice

**Fig. 6:**
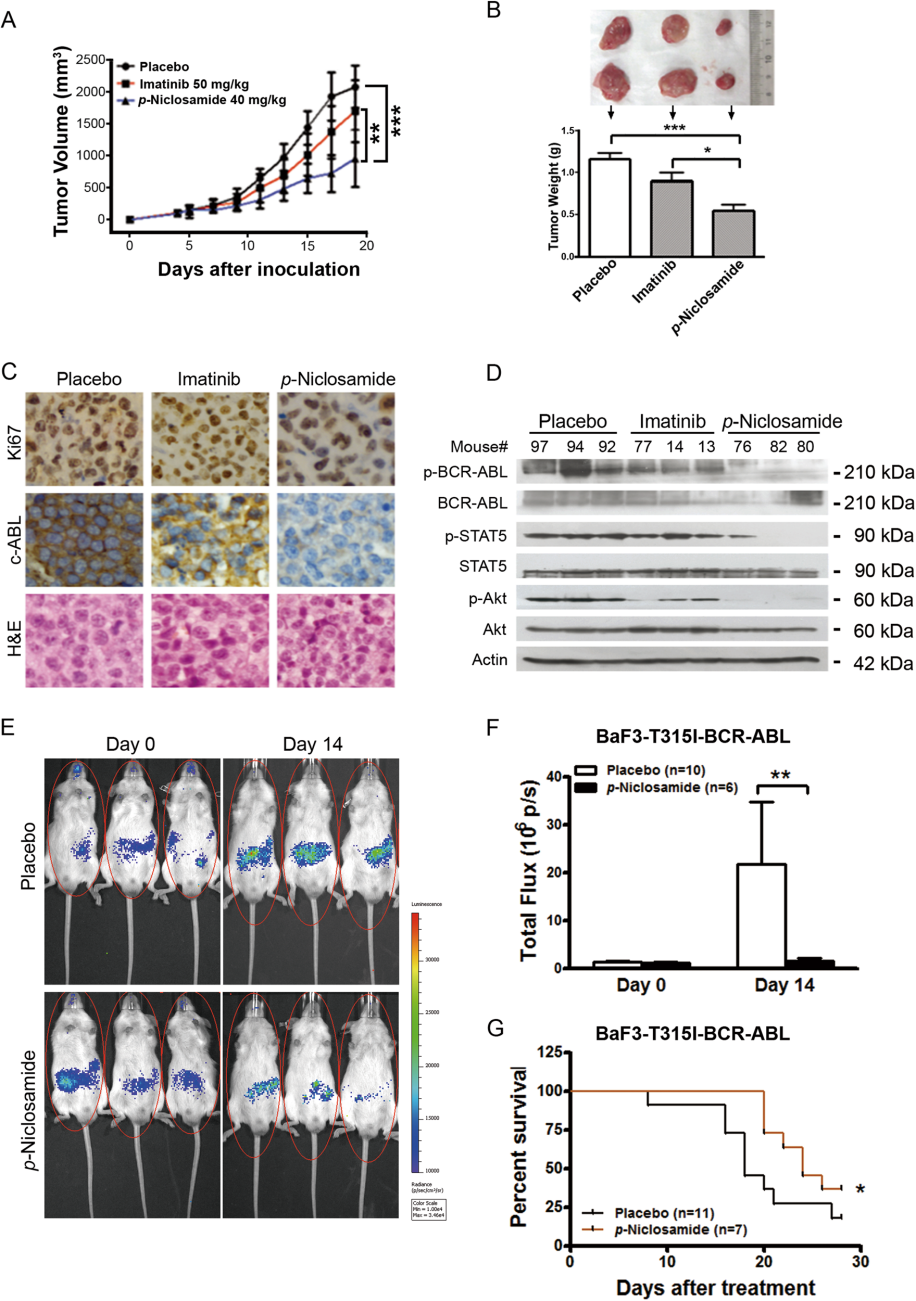
*p*-Niclosamide abrogates the growth of T315I-BCR-ABL-harboring cells in immunodeficient mice and prolongs the survival of allograft mice **a** Nude mice bearing KBM5-T315I xenograft tumors were treated with placebo (PBS), imatinib (served as a control) or *p*-niclosamide. Tumor volumes were plotted against days post CML cell inoculation. ***P < 0.0001, one-way ANOVA, compared placebo-treated or imatinib-treated mice with *p*-niclosamide-treated ones at the last time point. Data are mean ± 95% confidence intervals. **b** Tumor weights were assessed after two-week of treatment (bottom) and representative tumors of each group are shown (top). *P < 0.05, ***P < 0.0001, compared with Placebo, one-way ANOVA, *post hoc* intergroup comparisons. **c** Immunohistochemical analysis of Ki67 and c-ABL in KBM5-T315I xenografted tissues from mice sacrificed 19 days after inoculation of tumor cells. Hematoxylin and eosin (H&E)-stained sections of the same tissues were shown. **d** Western blotting analysis of BCR-ABL and its target molecules in xenografted tissues from the mice treated with placebo, *p*-niclosamide or imatinib. **e**, **f** Niclosamide inhibited the proliferation of BaF3-T315I-BCR-ABL-Luc cells in NOD/SCID mice. BaF3-T315I-BCR-ABL-Luc cells were injected into mice via tail vein, allowed to grow for 5 days, and then treated with placebo or *p*-niclosamide for 2 weeks. Representative photos of mice after treatment (**e**) and bar chart of *in vivo* luminescence signals (**f**) were shown. *P < 0.05, Student’s *t*-test. **g** Effect of *p*-niclosamide on survival of mice bearing BaF3-T315I-BCR-ABL-Luc cells. Kaplan–Meier survival curve of CML mice after treatment of *p*-niclosamide. *P = 0.0346, Log-Rank test

We assessed the *in vivo* effect of niclosamide on T315I-BCR-ABL cells using the nude mouse xenograft model. After treated for 2 weeks, 40 mg/kg of *p*-niclosamide substantially inhibited the growth of xenografted tumors while imatinib at 50 mg/kg barely inhibited the growth comparing with placebo, as shown in the tumor growth curve (Fig. 6a). The weights of tumors were significantly lower in *p*-niclosamide group than either imatinib or placebo (Fig. 6b). Immunohistochemical analysis showed decreased Ki67 staining further indicating the inhibition of growth of *p*-niclosamide on T315I-BCR-ABL cells (Fig. 6c).

We further examined the *in vivo* effect of *p*-niclosamide on BCR-ABL and downstream signaling. The levels of BCR-ABL were detected by immunohistochemical staining with anti-c-ABL (Fig. 6c) and further confirmed by Western blotting analysis (Fig. 6d). The downstream signaling molecules of BCR-ABL were assessed by Western blotting analysis with antibodies as indicated (Fig. 6d). The levels of phospho-STAT5 and phospho-Akt were decreased in the tumors from the mice treated by *p*-niclosamide with no alteration of their total levels, unlike the *in vitro* suppressive effect of niclosamide treatment on the total protein of STAT5 and Akt in KBM5-T315I cells (Fig. 6d), which might be associated with the single dose. In aggregate, these results suggest that *p*-niclosamide inhibits the growth of imatinib-resistant T315I-BCR-ABL cells *in vivo*.

### Niclosamide thwarts expansion of imatinib-resistant CML cells in NOD/SCID mice and prolongs the survival of these leukemia cell-bearing mice

To solidify the observed *in vivo* efficacy of *p*-niclosamide on CML, we employed the BaF3-T315I-BCR-ABL cells expressing firefly luciferase (BaF3-T315I-BCR-ABL-Luc) cells to visualize the growth by *in vivo* luminescence imaging with Xenogen IVIS Spectrum. BaF3-T315I-BCR-ABL-Luc cells were injected into NOD/SCID mouse via tail vein, allowed to grow for 5 days, and then treated with placebo or *p*-niclosamide for another 2 weeks. In the mice received placebo treatment, there was a significant increase in luminescence signal over 2 weeks (Fig. 6e, f). In contrast, the mice received *p*-niclosamide treatment displayed a decreased luminescence signal (Fig. 6e, f). Moreover, *p*-niclosamide significantly prolonged the survival of mice-bearing BaF3-T315I-BCR-ABL-Luc cells with an increase of median survival from 18 days (placebo group) to 24 days (*p*-niclosamide group) (Fig. 6g). Taken together, *p*-niclosamide thwarted expansion of imatinib-resistant CML cells in mice and prolonged the survival of mice bearing such cells.

## Discussion

**Fig. 7:**
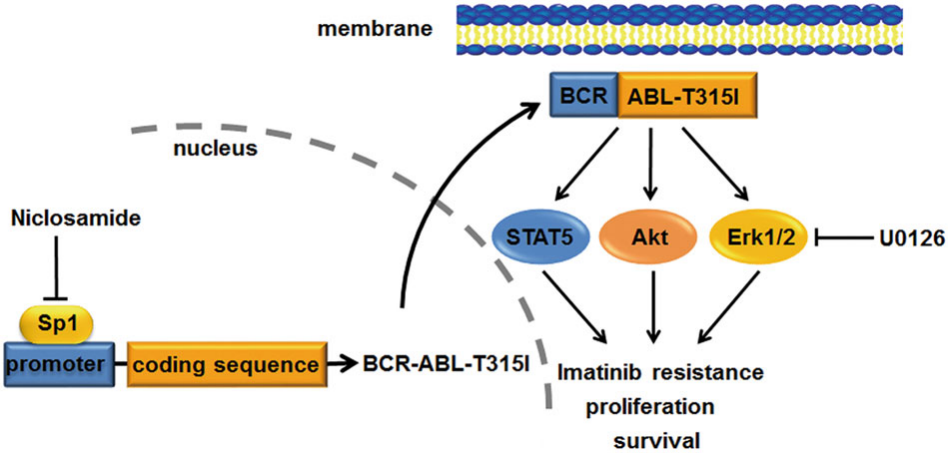
A proposed model of niclosamide activity against imatinib-resistant T315I mutation Niclosamide suppresses transcription of *BCR-ABL* fusion oncogene via disabling Sp1, resulting in the impairing of BCR-ABL signaling transduction

Ponatinib is among the limited options for imatinib-resistant CML patients harboring T315I-BCR-ABL. Albeit CML patients with the T315I-BCR-ABL mutation respond well to ponatinib^38^, an increased risk of thromboembolism with ponatinib seemed not inevitable^26^. In the present study, we found that Sp1 binds to the promoter of both WT- and *T315I*-*BCR-ABL* fusion genes. Niclosamide inhibited transcription of *BCR-ABL* gene by suppressing Sp1, and thereby abrogating downstream signaling (e.g., STAT5, Akt) of BCR-ABL (Fig. 7). Niclosamide decreased proliferation, induced apoptosis of imatinib-resistant KBM5-T315I cells as well as imatinib-sensitive KBM5 and K562 cells. The *in vivo* efficacy of niclosamide on imatinib-resistant cells harboring T315I-BCR-ABL was further validated with two types of mouse models.

We demonstrated that silencing Sp1 by dnSp1 increases sensitivity of CML cells to niclosamide, whereas overexpression of Sp1 attenuated apoptosis induced by niclosamide. Overexpression of Sp1 elevated the levels of BCR-ABL, while overexpression of dnSp1 led to declined expression of BCR-ABL. Furthermore, ChIP assay results revealed that Sp1 specifically and significantly enhanced the transcription of T315I- as well as WT-*BCR-ABL* gene (Fig. 1f). Our results are consistent with the report by Yang et al.^27^ that Sp1, in an EMSA assay, can bind to the promoter of WT-*BCR-ABL* fusion gene and that silencing Sp1 can diminish expression of WT-*BCR-ABL*. These findings may lay the foundation for endowing Sp1 an attractive therapeutic target in CML patients bearing *T315I-BCR-ABL*.

Results in the present study showed that niclosamide not only inhibited Sp1 expression, but also disturbed the Sp1 enrichment on *BCR-ABL* promoter. Both these mechanisms may confer the suppression of BCR-ABL expression and result in reduced proliferation and increased apoptosis in CML cells regardless of *T315I -BCR-ABL* mutation status. Niclosamide alone resulted in a significant decrease in Sp1 in both imatinib-sensitive (K562 and KBM5) and -resistant (KBM5-T315I) CML cells, suggesting that niclosamide may inhibit the transcription of *BCR-ABL* gene in a Sp1-dependent manner even in T315I-BCR-ABL-positive CML cells. Because miR-29b is a negative regulator of *Sp1* gene in acute leukemia cells^39,40^, the future work may need to identify whether miR-29b is involved in the niclosamide-mediated decrease in Sp1 in CML cells.

Niclosamide exerted inhibitory effect on transduction of BCR-ABL pathway as indicated by the reduced phospho-STAT5 and phospho-Akt in the niclosamide-treated CML cell regardless of BCR-ABL mutation status. Because the inhibitory mechanism against BCR-ABL pathway by niclosamide is distinct from the one of imatinib, our results imply less risk of cross-resistance of niclosamide with imatinib, and synergism between niclosamide and imatinib. Synergistic effect between niclosamide and imatinib in the abilities of inducing reduced proliferation and apoptotic cell death was, indeed, noted in imatinib-resistant and -sensitive CML cells. Given that Sp1 expression can be suppressed by the clinically available drug Bortezomib, an ubiquitin-proteasome inhibitor, in tumor cells^27^, our findings imply a rationale for a clinical trial of combination between imatinib and Bortezomib.

Unexpectedly, the levels of phospho-Erk1/2 at T202/Y204 were elevated with treatment of niclosamide. This might be due to the activation of a TORC1-PI3K feedback loop^35,41^. We speculated that combination of niclosamide and MEK inhibitor might be a potential approach to treat CML with T315I-BCR-ABL. Indeed, the combination of niclosamide with U0126 showed synergism in inhibiting cellular proliferation and inducing the apoptosis (Fig. 5). Niclosamide also potentiated the effects of TKIs in inducing cell death in cells harboring T315I mutation, while sparing the normal WBCs. Therefore, we underscored the potential therapeutic approach of combination between niclosamide and MEK inhibitors or TKIs.

The *in vivo* anti-leukemic effects appear encouraging. In the subcutaneous xenografts nude mouse model, *p*-niclosamide effectively inhibited the growth of xenografted tumor derived from KBM5-T315I cells in nude mice compared with the placebo- or imatinib-treated mice, suggesting the growth inhibition in T315I-BCR-ABL-bearing cells *in vivo*. Moreover, BCR-ABL signaling blockage by *p*-niclosamide as detected in tumor tissues from these xenografted mice supports the on-target effect of *p*-niclosamide. In a parallel model better mimicking CML, we performed the allograft of BaF3-BCR-ABL-T315I cells in NOD/SCID mice. The results showed that *p*-niclosamide prohibited the *in vivo* leukemic burden of BaF3-BCR-ABL-T315I cells and prolonged the survival of the mice-bearing BaF3-BCR-ABL-T315I cells, which further verified the *in vivo* anti-leukemic effectiveness of *p*-niclosamide in CML.

Niclosamide has minimal cytotoxicity against normal bone marrow and peripheral blood nucleated cells as reported previously^34,42^. Moreover, niclosamide can eradicate CML LSCs through disrupting the interplay between p65 and FOXM1/β-catenin, and prolonged the survival of CML mice^18^. In the present study, we further demonstrated the efficacy of niclosamide against imatinib-resistant BCR-ABL-T315I mutation with underlying mechanism that niclosamide inhibits expression of Sp1 and its enrichment on the promoter of either *WT-* or *T315I*-*BCR-ABL* fusion gene. In conclusion, our findings in these two studies warrant that niclosamide may represent a promising chemotherapeutic agent in the imatinib-resistant CML patient subpopulation harboring *T315I*-*BCR-ABL* and/or LSCs, and deserve a clinic trial.

## Materials and methods

### Chemicals and antibodies

Niclosamide (2′, 5-dichloro-4′-nitrosalicylanilide), annexin V-FITC, anti-actin (Cat#A5441) and mithramycin A (MMA) were from Sigma-Aldrich (St. Louis, MO). Niclosamide was dissolved in Dimethylsulfoxide (DMSO, Sigma, Shanghai, China) at a stock concentration of 10 mmol/L and stored in −20 °C. *p*-Niclosamide, a water soluble derivative of niclosamide, was designed by adding a phosphate group to niclosamide with diethyl phosphite. Imatinib was purchased from Novaris Pharmaceuticals (East Hanover, NJ). Antibodies against c-ABL (c-19, Cat#sc-887), apoptosis-inducing factor (AIF, H300, Cat#sc-13116), Bax (Cat#sc-493), and Mcl-1 (S-19, Cat#sc-819) were from Santa Cruz Biotechnology (Santa Cruz, CA). Antibodies against poly (ADP-ribose) polymerase (PARP, Cat#516639GR), caspase-3 (Cat#610322), active caspase-3 (Cat#557038), X-linked inhibitor of apoptosis protein (XIAP, Cat#610762), and cytochrome *c* (Cat#556432) were from BD Biosciences (San Jose, CA). Antibodies against caspase-8 (Cat#9746), -9(Cat#9508), phospho-BCR-ABL (Y245, Cat#2861), phospho-extracellular signal-regulated kinase 1/2 (phospho-Erk1/2, T202/Y204, Cat#9101), phospho-Akt (S473, Cat#9271), Akt (Cat#9272), and MEK inhibitor U0126 were from Cell Signaling Technology (Beverly, MA). Anti-phospho-STAT5 (Y694/Y699; clone 8-5-2, Cat#07-568), STAT5 (Cat#060553), Sp1 (Cat#07645) and Bcl-2 (Cat# 05-729) were from EMD Millipore (Billerica, MA). Anti-mouse immunoglobulin G and anti-rabbit immunoglobulin G horseradish peroxidase-conjugated antibodies were from Pierce Biotechnology (Rockford, IL).

### Cell culture

Cells bearing 210 kDa wild-type (KBM5) or T315I-BCR-ABL (KBM5-T315I) were grown in Iscove’s modified Dulbecco’s medium (IMDM, Invitrogen, Guangzhou, China) supplemented with 10% fetal bovine serum (FBS) as previously described^35,43^. 32D-BCR-ABL, 32D-T315I-BCR-ABL, K562 and BaF3-T315I-BCR-ABL-Luc cells were incubated in RPMI-1640 (Invitrogen, Guangzhou, China) with 10% FBS. 293T cells were cultured in Dulbecco’s modified Eagle's medium  (DMEM) supplemented with 10% FBS. Cells were incubated at 37 °C and in water vapor-saturated air with 5% CO_2_ at one atmospheric pressure. All the cell lines were tested and authenticated by using short tandem repeat (STR) matching analysis of cells every 6 months. No cross-contamination of other human cells was found in all six lines of cells. Peripheral blood samples were obtained from healthy adult donors in Zhongshan Ophthalmic Center, Sun Yat-sen University. White blood cells were isolated in Ficoll. The study followed institutional guidelines and the Declaration of Helsinki principles.

### Luciferase assay

293T cells were transfected with reporter plasmids encoding *BCR-ABL*-Luc (0.5 μg), TOPflash (0.5 μg), FOPflash (0.5 μg), pEBGN-Sp1 (0.5 μg)^44^, pEBGN-dnSp1 (0.5 μg)^44^, and pEF*Renilla*-luc (10 ng) by Lipofect AMINE 2000 (Invitrogen, CA). After 24 h, cells were exposed to different concentrations of niclosamide for 24 h or a fixed concentration of niclosamide for various durations. Luciferase activities were measured with dual-luciferase assay kit (Promega, Madison, WI), as previously described^45^. Results were expressed by normalizing the activity of targeted gene-dependent firefly luciferase to that of *Renilla* luciferase.

### Real-time quantitative PCR

Total mRNA extracted with TRIzol reagent (Invitrogen, CA) was reverse-transcribed into cDNA with maxima first strand cDNA synthesis kit (Thermo Fisher). Sixty nanogram of cDNA was used for real-time quantitative PCR (qRT-PCR) with the SYBR Premix Ex Taq Kit (Takara, Dalian, China) according to the manufacturer’s recommended protocol. The specific primers for the amplification cDNA were as follows: *BCR-ABL*, forward, 5′-TCCACTCAGCCACTGGATTTAA-3′; reverse, 5′-TGAGGCTCAAAGTCAGATGCTACT-3′; *Sp1*, forward, 5′-ACCTGAGGAGACATCTACAC-3′; reverse, 5′-GGAGGCTACAGACTACATTG-3′; *18S*, forward, 5′- AAACGGCTACCACATCCAAG -3′; reverse, 5′- CCTCCAATGGATCCTCGTTA -3′.

### Chromatin immunoprecipitation (ChIP) assay

K562 and KBM5-T315I cells were treated with or without 2.0 μM niclosamide for 24 h, and then 1 × 10^7^ cells were collected and prepared with ChIP kit (Cat# 17–371, EMD Millipore) according to the manufacturer’s instructions^17^. In brief, cells were fixed with 1% formaldehyde to covalently crosslink proteins to DNA and harvested in SDS lysis buffer containing protease inhibitor cocktail. Then cross-linked DNA was sonicated into 200–600 bp followed by preclearance with 60 µL of protein G agarose for 1 h. The supernatant was then immunoprecipitated with 2.0 µg of Sp1 antibody or normal rabbit IgG at 4 °C overnight for purification of associated DNA. Protein–DNA complexes were pelleted, washed, eluted and then reversed to free DNA with proteinase K by overnight incubation at 65 °C. DNA was purified using spin columns. Purified DNA was then subjected to qRT-PCR with the SYBR Premix Ex Taq Kit (Takara, Dalian, China) according to the manufacturer’s recommended protocol. The amplification procedures were: 5 min at 94 °C for initial denaturation, 30 s at 94 °C for denaturation, 30 s at 60 °C for annealing, 30 s at 72 °C for extension, repeat for 40 cycles, 72 °C for 7 min for final extension. The primers for *BCR-ABL* promoter were: forward, 5′-CCGCCTGGCTCCGTCATCC-3′; reverse, 5′-CCTCGGACGCTAAGCTCAGCC-3′.

### Cell viability assay

Cell viability was determined by MTS assay (CellTiter 96 Aqueous One Solution Cell Proliferation assay; Promega, Shanghai, China). Briefly, 100 μL cells (2 × 10^5^/mL) were inoculated in 96-well plate at different concentrations of niclosamide for 72 h. Four hours before culture termination, 20 μL MTS solution was added to each well. The absorbance was read on a 96-well plate reader at 490 nm. The drug concentration resulting in 50% growth inhibition (IC_50_) was determined in Microsoft excel sheet by linear regression.

### Soft agar colony-formation assay

KBM5, KBM5-T315I, and K562 cells were treated with increasing concentrations of niclosamide or diluent (DMSO, control) for 48 h, then washed with phosphate-buffered saline (PBS), and seeded in IMDM containing 0.3% agar and 20% FBS in the absence of drug treatment. After incubation for 10~14 days at 37 °C, colonies containing more than 50 cells were counted^46^.

### Preparation of whole-cell lysates and cytosolic fractions

Control or niclosamide-treated cells were collected by centrifugation, and then rinsed with PBS. The whole-cell lysates were then lysed with radioimmunoprecipitation assay buffer (1 × PBS, 1% NP-40, 0.5% sodium deoxycholate, 0.1% SDS) containing freshly added 10 mmol/L glycerophosphate, 1 mmol/L Na_3_VO_4_, 10 µg/mL pepstatin A, 5 µg/mL aprotinin, 10 µg/mL leupeptin, 10 mmol/L NaF, and 1 mmol/L phenylmethylsulfonyl fluoride^47^. The cytosolic fractions were prepared with digitonin extraction buffer (10 mmol/L PIPES pH 6.8, 0.015% digitonin, 300 mmol/L sucrose, 100 mmol/L NaCl, 3 mmol/L MgCl_2_, 5 mmol/L EDTA) to detect the levels of cytochrome *c* and AIF in the cytosol, as described previously^47^.

### Flow cytometer analysis

#### Cell cycle analysis

Cells were treated with different concentrations of niclosamide for 24 h, then harvested, washed with PBS and fixed with 66% ethanol overnight. Then cells were stained with 50 μg/mL propidium iodide and 10 μg/mL RNase in PBS solution for 30 min at the room temperature. DNA content was analyzed by flow cytometer at the emission wavelength of 488 nm.

#### Annexin V-FITC/PI dual staining analysis

Cells were cultured in the presence of niclosamide for 48 h or 2 μM niclosamide at different durations, and then harvested, washed with PBS and incubated in binding buffer (annexin V binding buffer, BD pharmingen) with 0.3% annexin V-FITC for 20 min at room temperature. Cells were washed and resuspended in binding buffer. Propidium iodide was added just before flow cytometer analysis.

#### Mitochondrial transmembrane potential measurement

KMB5 and KBM5-T315I cells were incubated with 2.0 μmol/L niclosamide for the indicating durations. K562 cells were treated with 10.0 μmol/L niclosamide for different durations. All cells were incubated with Mito Tracker probes (CMXRos and MTGreen, Eugene, OR) followed by flow cytometer analysis for the changes in inner mitochondrial transmembrane potential as previously described^34^.

### In vivo anti-tumor effect of *p*-niclosamide

For nude mouse xenograft experiments: Male *nu/nu* BALB/c mice were purchased from Slac Laboratory Animal Co (Shanghai, China). KBM5-T315I cells (3 × 10^7^ in 200 μL serum-free medium) were inoculated subcutaneously on the flanks of 4~6-week-old mice. Tumors were measured every other day with calipers. Tumor volumes were calculated by the following formula: *a*^2^ × *b* × 0.4, where *a* is the smallest diameter and *b* is the diameter perpendicular to *a*. Four days after cells inoculation, when tumors were palpable, mice were randomized to receive treatment with placebo (PBS, i.p., twice a day), imatinib (50 mg/kg, oral gavage, once a day) or *p*-niclosamide (40 mg/kg, i.p., twice a day) for ~2 weeks. After mice were euthanized, xenografts were dissected, weighted, and preserved.

For* in vivo* luminescence imaging, BaF3-T315I-BCR-ABL-Luc cells coexpressing firefly luciferase (2 × 10^6^ cells in 200 μL PBS) were intravenously injected into NOD/SCID mice. The proliferation of BaF3-T315I-BCR-ABL-Luc cells in mice was monitored through in vivo luminescence imaging with Xenogen IVIS Spectrum (Baltimore, MD). Briefly, mice were intraperitoneally injected with 150 mg/kg D-luciferin potassium solution and anesthetized with halothane. The *in vivo* luminescence images of mice were taken five minutes later. Mice were than randomized to different treatment groups (placebo, PBS, i.p., twice a day; *p*-niclosamide, 40 mg/kg, i.p., twice a day) based on the magnitude of the luminescence signal and treated for 2 weeks. The body weight was recorded every two days. The proliferation of BaF3-T315I-BCR-ABL-Luc cells was monitored by *in vivo* luminescence imaging at the indicated time points. Survival of the mice were recorded and analyzed with Kaplan–Meier method using GraphPad Prism 5.0 (San Diego, CA).

Mice were kept under specific pathogen-free conditions in Sun Yat-sen University animal care facility. All animal studies were approved by the Sun Yat-sen University Institutional Animal Care and Use Committee.

### Immunohistochemical staining

Formalin-fixed tissues were embedded in paraffin and sectioned. Tumor xenograft sections (4.0 μm) were immunostained with Ki67 and c-ABL antibodies using the MaxVision kit (Maixin, Fuzhou, China) according to the manufacturer’s instructions^37^.

### Statistical analysis

GraphPad Prism 5.0 (San Diego, CA) was used for statistical analysis. All experiments were carried out at least three times, and results were expressed as mean ± 95% confidence intervals unless otherwise stated. Comparison between two groups was analyzed by Student’s *t*-test and between more than two groups by one-way ANOVA with post hoc comparison by Tukey test. *P* < 0.05 was considered statistically significant.

## Electronic supplementary material

Supplementary Figure S1

Supplementary Figure S2
