# Supplementary material for: Anthelmintic niclosamide suppresses transcription of BCR-ABL fusion oncogene via disabling Sp1 and induces apoptosis in imatinib-resistant CML cells harboring T315I mutant
Source: Cell Death Dis. 2018 Jan 22;9(2):68. doi: 10.1038/s41419-017-0075-7 (PMC5833368; doi:10.1038/s41419-017-0075-7)
Supplement: Supplementary file 1 — Supplementary Figure S1 [file 41419_2017_75_MOESM1_ESM.pdf]

Supplementary Figure S1

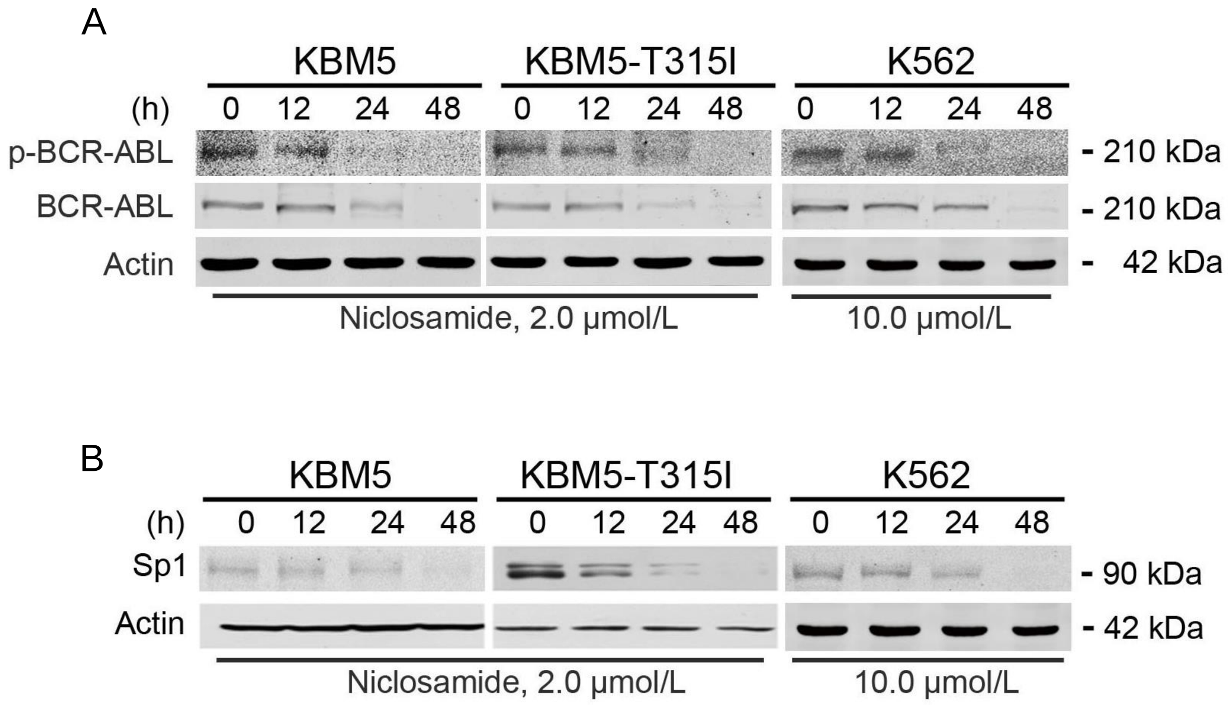

Supplementary Figure S1. Niclosamide suppresses transcription of *BCR-ABL* gene by lowering transcriptional factor Sp1 in CML cells harboring either wild type- or T315I-BCR-ABL.

A, KBM5 cells harboring wild type or T315I-BCR-ABL and K562 cell were exposed to niclosamide as indicated for different durations, and then analyzed by Western blotting. Actin served as a loading control for lanes above. B, Sp1 levels were downregulated in CML cells. KBM5, KBM5-T315I and K562 cells were treated with the indicating niclosamide for 48 h and subjected to Western blotting analysis.
