# Supplementary material for: Anthelmintic niclosamide suppresses transcription of BCR-ABL fusion oncogene via disabling Sp1 and induces apoptosis in imatinib-resistant CML cells harboring T315I mutant
Source: Cell Death Dis. 2018 Jan 22;9(2):68. doi: 10.1038/s41419-017-0075-7 (PMC5833368; doi:10.1038/s41419-017-0075-7)
Supplement: Supplementary file 2 — Supplementary Figure S2 [file 41419_2017_75_MOESM2_ESM.pdf]

Supplementary Figure S2

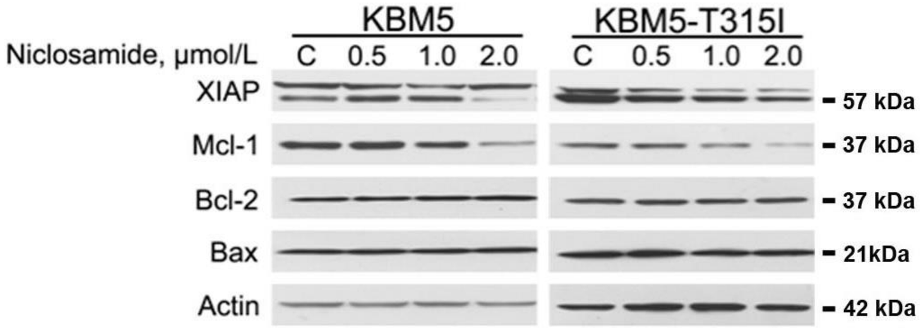

Supplementary Figure S2. Niclosamide decreased the level of Mcl-1 and XIAP.

Niclosamide decreased the level of Mcl-1 and XIAP in a dose-dependent manner. KBM5 and KBM5-T315I cells with niclosamide treatment were subjected to Western blotting analysis.
